# Identification of MEDAG as a Hub Candidate Gene in the Onset and Progression of Type 2 Diabetes Mellitus by Comprehensive Bioinformatics Analysis

**DOI:** 10.1155/2021/3947350

**Published:** 2021-02-25

**Authors:** Jing Yang, Ping Yu

**Affiliations:** ^1^Department of Infectious Diseases, Sir Run Run Shaw Hospital, Zhejiang University School of Medicine, Hangzhou 310020, China; ^2^Department of Endocrinology, The Third People's Hospital of Hangzhou, Hangzhou 310009, China

## Abstract

**Objectives:**

We conducted the present study to identify novel hub candidate genes in the pathogenesis of type 2 diabetes mellitus (T2DM) and provide potential biomarkers or therapeutic targets for dealing with the disease.

**Methods:**

We conducted weighted gene coexpression network analysis on a series of the expression profiles of the pancreas islet of T2DM patients obtained from the Gene Expression Omnibus database to construct a weighted coexpression network. After dividing genes into separated coexpression modules, we identified a T2DM-related module using Pearson's correlation analysis. Then, hub genes were identified from the T2DM-related module using the Maximal Clique Centrality method and validated by correlation analysis with clinical traits, differentially expressed gene analysis, validation in other datasets, and single-gene gene set enrichment analysis (GSEA).

**Results:**

Genes were divided into 16 coexpression modules, and one module was identified as a T2DM-related module. Four hub candidate genes were identified, and MEDAG was a novel hub candidate gene. The expression level of MEDAG was positively correlated with hemoglobin A1c (HbA1c) and was evidently overexpressed in the pancreas islet tissue of T2DM patients compared with normal control. Analyses on two other datasets supported the results. GSEA verified that MEDAG plays essential roles in T2DM.

**Conclusions:**

MEDAG is a novel hub candidate of T2DM, and its irregular expression in the pancreas islet plays vital roles in the pathogenesis of T2DM. MEDAG is a potential target of intervention in the future for the treatment of T2DM.

## 1. Introduction

Diabetes mellitus (DM) is a prevalent metabolic disease leading to multiple complications, high mortality, and heavy economic burdens. Globally, one in 11 adults are suffering from DM, 90% of whom are type 2 diabetes mellitus (T2DM) patients [1].

The most significant pathophysiological characteristics of T2DM are decreases in insulin secretion capacity and peripheral insulin sensitivity [2]. Islet *β*-cell dysfunction is the most important cause of T2DM, and researchers have developed various medications to deal with T2DM accordingly. However, as the pathogenesis of T2DM has not been completely clarified, the disease has not been conquered yet. Thus, it is an urgent mission for us to identify more hub candidate genes in T2DM for exploring the mechanism and developing more therapeutic approaches for T2DM.

With the rapid development of gene chip and next-generation sequencing techniques, as well as the popularization of public databases, bioinformatics analysis has become a new approach for identifying vital molecular components in the pathogenesis of diseases. Weighted gene coexpression network analysis (WGCNA) is a bioinformatics analysis tool based on the hypothesis that the biological expression pattern of genes obeys the characteristics of a scale-free network, and the central factors in the network would be more easily found out when the coexpression relationships are weighted [3]. The technique has been broadly used to explore new hub candidate genes, biomarkers, or therapeutic targets in various kinds of diseases including acute diseases, chronic diseases, infectious diseases, and tumors [4–7].

In the present study, we performed WGCNA on a series of expression profiles of pancreas islet tissue of T2DM patients and normal control obtained from the Gene Expression Omnibus (GEO) public database to construct a weighted coexpression network. After confirming a T2DM-related coexpression gene module, we performed systematical analyses on it and identified hub candidate genes of T2DM. Then, we validated the functions of known hub genes and explored the functions of the newly identified hub genes. Depending on the identification of the hub gene and exploration of its potential roles in the dysfunction of insulin secretion in the pancreas islet, we expect to provide novel insights for explaining and treating the disease.

## 2. Materials and Methods

### 2.1. Data Collection

**Figure 1:**
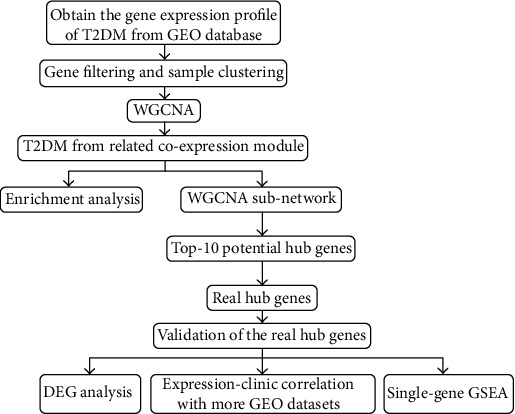
Complete procedures of our research.

In the beginning, we exhibit the design and overall procedures of the present research in a flowchart (Figure 1). We searched the GEO database (https://www.ncbi.nlm.nih.gov/geo/) with the keyword “type 2 diabetes mellitus”. Of all expression profiles of human pancreatic islet, we selected GSE41762 [8] for WGCNA as it had relative more T2DM samples than the other datasets (the dataset of GSE41762 contains 20 T2DM samples and 56 normal samples in all). For subsequently validating the conclusions drawn in WGCNA, we collected another two datasets of pancreatic islet from the GEO database, including GSE38642 (containing 31 normal samples, 10 IGT samples, and 10 T2DM samples) [9] and GSE50397 (containing 68 normal samples, 29 IGT samples, and 16 T2DM samples) [10]. We downloaded the txt files of expression profiles of the three datasets from the GEO database. The expression level of genes has already been log2 transferred, and all the three datasets were detected by the gene chip technique based on the microarray platform of GPL6244.

### 2.2. Probe Annotation

Probe annotation was conducted to convert the signal intensity quantified by probes into the mRNA expression levels of genes. We performed this procedure under R environment with the annotation platform file of GPL6244. If a probe pointed to more than one gene symbol, we took the first gene symbol in the platform file as the detected gene. If a gene was detected by more than one probe, we calculated the average of the probes as the expression level of the gene.

### 2.3. Gene Filtering

The complete expression profile of GSE41762 contains the expression level of 23304 genes after probe annotation. Firstly, we excluded genes with missing values from our study. Then, as genes of low variance are regarded as noises for WGCNA and may affect the results of the analyses, we filtered these genes and reserved genes of top 5000 variance for subsequent bioinformatics analyses.

### 2.4. Sample Clustering

We conducted sample cluster analysis using a hierarchical clustering method included in the WGCNA package under R environment [3] to evaluate the quality of the data.

### 2.5. Construction of the Weighted Gene Coexpression Network and Division of Coexpression Modules

Rather than simply estimating the coexpression relationships between genes with Pearson's correlations, WGCNA is aimed at constructing a weighted network on the basis of Pearson's coexpression network in order to emphasize the pivotal relationships and weaken the others.

Firstly, we evaluated the correlation relationships of pairwise genes using Pearson's correlation analysis and constructed Pearson's correlation matrix. Secondly, we transformed Pearson's correlation matrix into an adjacency matrix (also known as scale-free network) with an appropriate soft thresholding value (*β* value) for conducting exponentiation. A bigger *β* value generally means a higher scale-free fit index but worse mean connectivity of the whole network. To balance the two factors, we decided the *β* value with the highest mean connectivity when the scale-free fit index reached 0.85 as the most proper one and then constructed the scale-free network according to the selected *β* value. Finally, to further highlight the pivotal genes, we took indirect connections into consideration by converting the scale-free network into a topological overlap matrix (TOM).

After reevaluating the coexpression relationships through constructing a weighted gene coexpression network, we divided all genes into coexpression modules by average linkage hierarchical clustering based on the TOM-based dissimilarity measure. Thus, genes sharing related functions (coexpression relationships) were divided into the same module and genes carrying out separated functions would be divided into different modules.

Additionally, we merged modules of high similarity (higher than 0.75) with each other together.

### 2.6. Identification of Clinical-Related Gene Modules

The owner of GSE41762 had provided some clinical characteristics listed as follows: disease status (diabetes patients or normal control), hemoglobin A1c (HbA1c), sex, age, and body mass index (BMI). Our study mainly focused on the hub genes related to T2DM, so we analyzed the correlations between modules and disease status. We selected modules of significant correlations (positive or negative correlations) with T2DM for the following analyses.

Here, we assessed the reliability of the clinical-related modules with the correlation between gene significance (GS) and module membership (MM) for genes in a module [3]. GS is defined as Pearson's correlation of a gene with a clinical trait, and MM represents Pearson's correlation of a certain gene in a module with the module eigengene (the first principal component of a given module) of the module. If the correlation coefficient between GS and MM is high in a module, we could conclude that the genes in the module contribute to the module and the clinical trait at the same time to a great extent, and the module is worthy of excavation of hub genes.

### 2.7. Enrichment Analyses

Enrichment analyses are conducted to explore the biological functions of a cluster of genes. The most frequently used methods include Gene Ontology (GO) [11] enrichment analysis and Kyoto Encyclopedia of Genes and Genomes (KEGG) [12] pathway enrichment analysis. We conducted enrichment analyses on genes in clinic-related modules, respectively, with the clusterProfiler package [13] under R environment. The cut-off criteria for statistically significantly enriched terms were set as *p* < 0.01 and Benjamin-Hochberg-adjusted *p* value < 0.05. As samples in our study were islet tissue, so if the results of enrichment analyses show clues of impaired function of the pancreas islet, we could conclude that genes in the module were involved in the pathogenesis of T2DM.

### 2.8. Candidate Hub Gene Identification

As genes in the T2DM-related module were regarded as participating in the pathogenesis of T2DM, we extracted all genes as well as their weighted coexpression relationships with each other and constructed a subnetwork. Then, we analyzed the centrality of each gene in the subnetwork by the Maximal Clique Centrality (MCC) [14] method with cytoHubba (a plug-in of Cytoscape software [15]) and introduced the conception of the MCC value as a criterion for evaluating the centrality of genes in the subnetwork. The higher the MCC value is, the more significant the genes are. We confirmed genes with top 10 MCC values as potential hub candidate genes in the pathogenesis of T2DM.

### 2.9. Identification of a Real Hub Gene by Correlation Analysis between Hub Genes and Clinical Traits

If the expression levels of selected hub genes are related to specific clinical features of the disease, we could conclude that the functions of the genes play important roles in the progression of the disease. During the disease course of T2DM, HbA1c is a vital indicator for the severity of the disease, so we conducted Pearson's correlation analysis between the expression levels of top 10 potential hub genes (with top 10 MCC values) and HbA1c to identify the real hub genes in T2DM. The criteria for significant correlation were set as coefficient index (cor) > 0.4 and *p* value < 0.01.

### 2.10. Validation of a Hub Gene of T2DM with External GEO Datasets

To further enhance the credibility of the conclusions, we validated the correlation between hub genes and HbA1c with two other datasets of T2DM obtained from the GEO database (GSE38642 and GSE50397).

### 2.11. Validation of a Hub Gene by Differentially Expressed Gene Analysis

The irregular expression levels of hub genes in certain tissues usually make up the key factor of pathogenesis of the disease. We conducted differentially expressed gene (DEG) analysis on the T2DM-related coexpression module to validate our hub gene. DEGs were defined as genes with ∣log fold change | >0.5 in a diabetes pancreas islet compared with normal control and statistical significance of *p* < 0.01. DEG analysis was performed in GSE41762 using the most frequently used R package for DEG analysis known as limma [16].

### 2.12. Function Analysis for Real Hub Genes by Single-Gene Gene Set Enrichment Analysis

Single-gene gene set enrichment analysis (GSEA) [17] was an approach for exploring the roles of a single gene in certain disease with the expression profile of the disease. If the aberrant expression level of a certain gene is vital for a given disease, we have reasons to believe that the expression patterns of other genes differ a lot between high-expression and low-expression groups of the given gene. Thus, we divided the samples (including T2DM and normal) into high- and low-expression groups of a certain real hub gene, so that we could conduct GSEA on the hub genes, respectively, to explore their potential functions in the disease. We performed GSEA using its desktop tool [18] with a cut-off criterion of *p* < 0.05.

## 3. Results

### 3.1. Data Preprocessing

**Figure 2:**
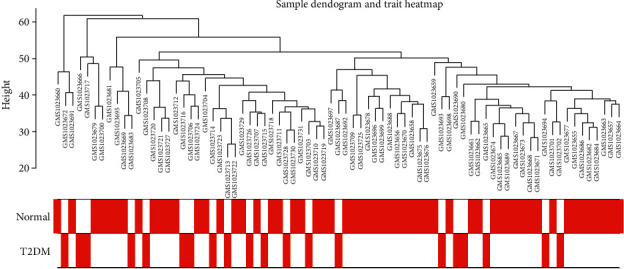
Sample cluster of all samples including T2DM and normal control.

We got the expression profiles containing the expression levels of 23304 genes after probe annotation and retained 5000 genes for subsequent analyses after gene filtering. Result of sample clustering (Figure 2) indicated that most T2DM samples gathered in the left branches of the clustering tree, revealing relative satisfactory intra-group consistency and inter-group difference of the samples.

### 3.2. Construction of the Weighted Gene Coexpression Network and Division of Coexpression Modules

**Figure 3:**
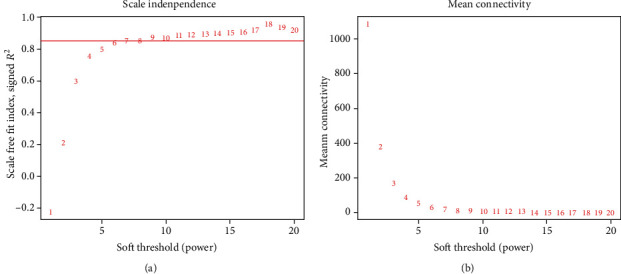
Decision of proper soft thresholding value (*β* value) for constructing a scale-free network: (a) scale-free fix index of each *β* value (the abline represents a *β* value of 0.85) and (b) mean connectivity of each *β* value.

The expression matrix of the 5000 genes was finally transformed into a weighted gene coexpression network according to the three procedures mentioned above. The most essential procedure was the selection of the *β* value, in which we decided 7 as the proper one as it gave the network highest mean connectivity when the scale-free fit index was above 0.85 (Figure 3).

**Figure 4:**
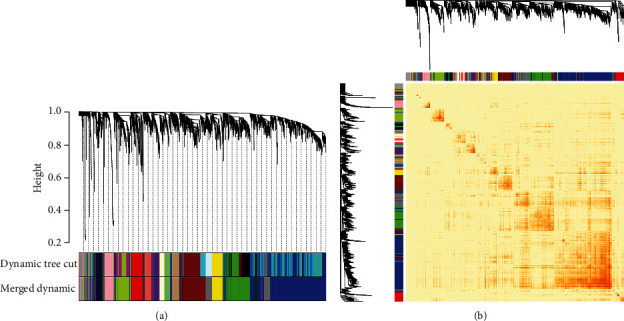
Construction of the weighted coexpression network. (a) A dendrogram of all genes and the division of the modules (before and after merging). (b) Adjacency heatmap of all genes involved in the coexpression network. The depth of the color represents the intensity of coexpression relationships.

After the construction of the weighted network, all 5000 genes were divided into 16 coexpression modules, and the modules were provisionally labeled and named by their colors (Figure 4(a)). Genes in grey modules were not belonging to any coexpression modules.

The TOM adjacency correlations of all genes are visualized as an adjacency heatmap (Figure 4(b)), revealing that the genes were mainly coexpressed with genes in the same module and had weak correlations with genes in different modules. The adjacency analysis of genes indicated satisfactory accuracy of module division.

### 3.3. Module-Clinic Correlation Analysis and Identification of Clinic-Related Module

**Figure 5:**
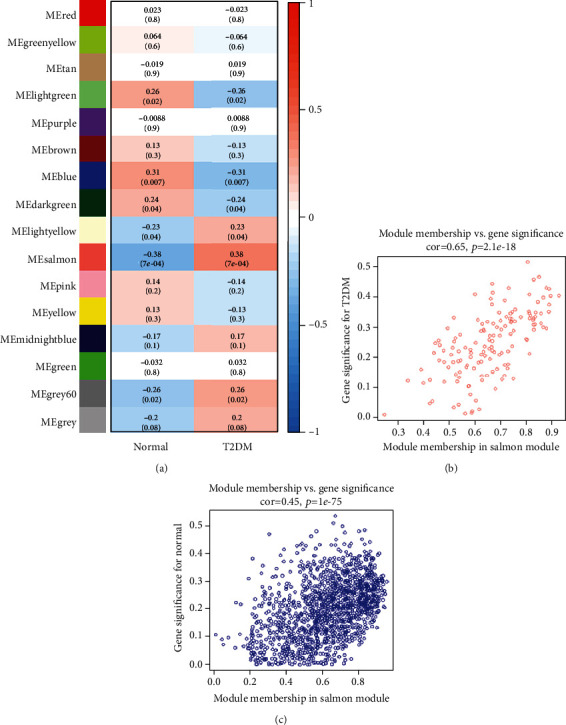
Identification and validation of clinic-related modules. (a) Correlation analysis of all modules with clinical characteristics. Correlation coefficient and statistical significance of each module are given in each cell, and the color represents the correlation coefficient. (b) Correlation of GS and MM in the salmon (T2DM-related) module. (c) Correlation of GS and MM in the blue (normal-related) module.

We assessed Pearson's correlation between modules and clinical traits (T2DM and normal control) to find out modules with significant influence on the disease (Figure 5(a)). The results showed that the “salmon module” (including 142 genes) was significantly related to the clinical trait of T2DM (cor = 0.38, *p* = 7*E* − 4), and the blue module was significantly related to the clinical trait of the normal control (cor = 0.31, *p* = 0.007). Genes in the salmon module were deemed as playing a vital role in the pathogenesis of T2DM, and genes in the blue module were essential for keeping regular biological function in the normal control. So we renamed the modules as T2DM-related module and normal-related module, respectively, for subsequent analyses.

The correlation between GS and MM for all genes in T2DM-related and normal-related modules is shown in Figures 5(b) and 5(c), and the correlation coefficient and statistical significance were cor = 0.65 and *p* = 2.1*E* − 18 and cor = 0.45 and *p* = 1*E* − 75, respectively, demonstrating that the genes in the modules were significantly correlated with the module eigengene and clinical characteristics simultaneously.

Thus, we decided that both T2DM-related and normal-related modules were worthy of further analyses.

### 3.4. Enrichment Analyses on Clinic-Related Modules

**Figure 6:**
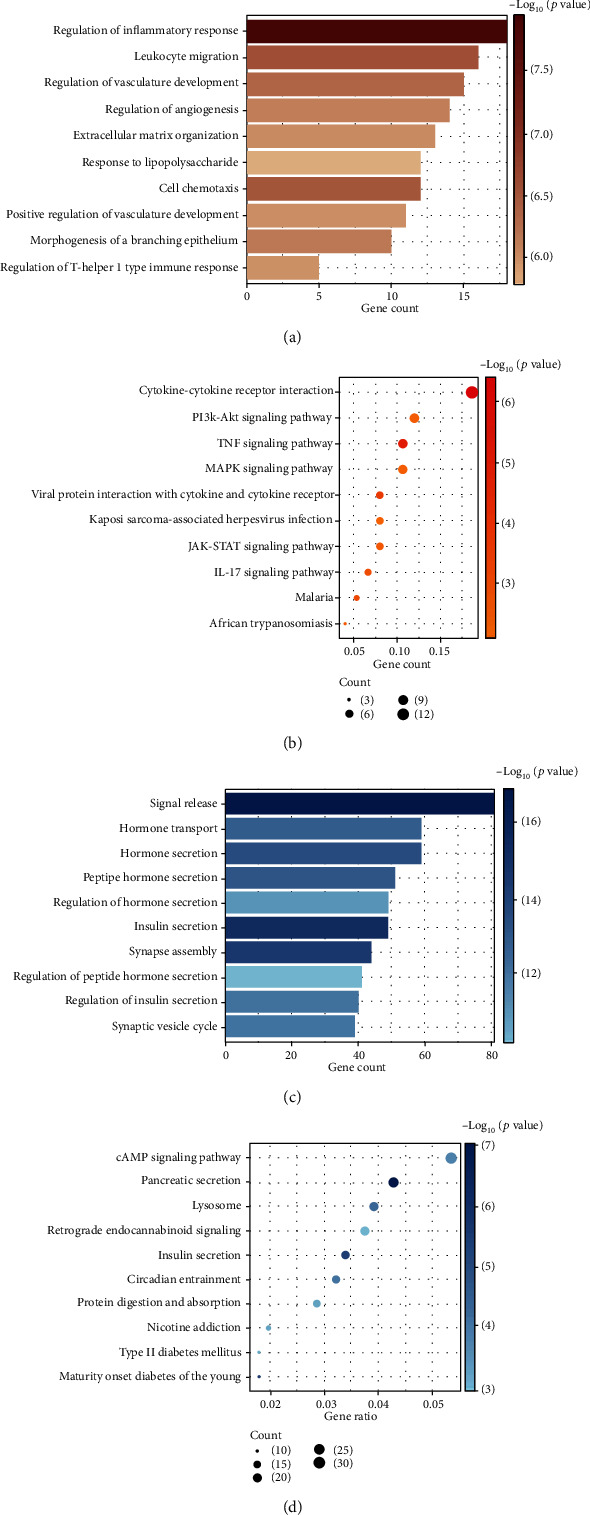
Results of enrichment analyses: (a) top 10 significant terms of GO-BP enrichment analysis of the T2DM-related module; (b) top 10 significant terms of KEGG enrichment analysis of the T2DM-related module; (c) top 10 significant terms of GO-BP enrichment analysis of the normal-related module; (d) top 10 significant terms of KEGG enrichment analysis of the normal-related module.

We performed GO and KEGG enrichment analyses on genes in two clinic-related modules, respectively, to clarify the main dysfunction of biological processes in T2DM. We exhibited some of the top-enriched GO-BP terms and KEGG terms in Figure 6 and provided the complete results of significantly enriched terms in Supplement Table 1.

For the T2DM-related module, GO enrichment analysis indicated the participation of inflammatory response and immune response in the pathogenesis of T2DM. Top terms of GO-BP are listed such as “regulation of inflammatory response” (gene count = 18, *p* = 1.15*E* − 08), “leukocyte migration” (gene count = 16, *p* = 2.80*E* − 07), and “regulation of T-helper 1 type immune response” (gene count = 5, *p* = 1.08*E* − 06). KEGG enrichment analysis provided similar results, such as “cytokine-cytokine receptor interaction” (gene count = 14, *p* = 4.23*E* − 07), “TNF signaling pathway” (gene count = 8, *p* = 8.58*E* − 06), and “IL-17 signaling pathway” (gene count = 5, *p* = 0.002).

For the normal-related module, GO enrichment hinted the dysfunction of insulin secretion. Enriched terms are listed as “insulin secretion” (gene count = 49, *p* = 8.33*E* − 16), “hormone transport” (gene count = 59, *p* = 2.20*E* − 13), and “signal release” (gene count = 81, *p* = 1.52*E* − 17). Results of KEGG enrichment were similar, for example, “pancreatic secretion” (gene count = 24, *p* = 8.31*E* − 08), “insulin secretion” (gene count = 19, *p* = 5.11*E* − 06), and “type II diabetes mellitus” (gene count = 10, *p* = 0.001).

### 3.5. Candidate Hub Gene Identification

**Figure 7:**
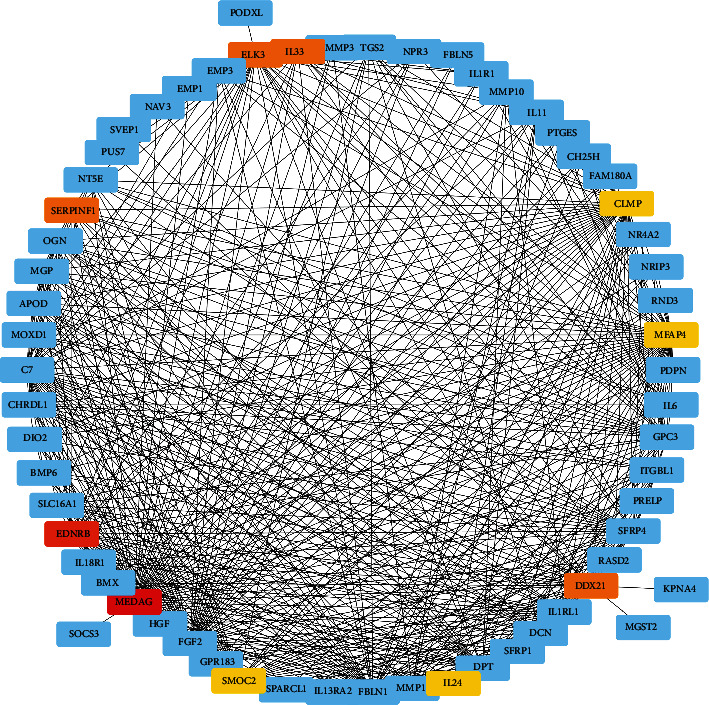
MCC analysis of genes in the T2DM-related module (the red and yellow cells are top candidate genes).

We here aimed to identify a hub candidate gene in the pathogenesis of T2DM, so we constructed a subnetwork of WGCNA with all genes and their weighed coexpression correlation coefficients in the T2DM-related module. By MCC analysis (Figure 7), we decided 10 potential hub genes in the pancreas islet blamed for T2DM: *MEDAG*, *EDNRB*, *DDX21*, *SERPINF1*, *ELK3*, *IL33*, *SMOC2*, *IL24*, *CLMP*, and *MFAP4*. These 10 genes had the highest MCC value in the subnetwork and thus had the strongest coexpression relationships with other genes in the whole subnetwork.

### 3.6. Identification of Real Hub Genes

We analyzed the correlation between the expression levels of the 10 potential hub genes and HbA1c, respectively, using Pearson's correlation analysis to identify the real hub genes involved in T2DM. According to the criteria mentioned before, *MEDAG* (cor = 0.404, *p* = 0.001), *SERPINF1* (cor = 0.532, *p* = 6.98*E* − 6), *IL33* (cor = 0.451, *p* = 2.02*E* − 4), *SMOC2* (cor = 0.422, *p* = 5.66*E* − 4), *IL24* (cor = 0.438, *p* = 3.35*E* − 4), *CLMP* (cor = 0.420, *p* = 6.06*E* − 4), and *MFAP4* (cor = 0.440, *p* = 3.03*E* − 4) are identified as real hub genes for T2DM.

### 3.7. Validation of a Hub Gene with External GEO Datasets of T2DM

**Figure 8:**
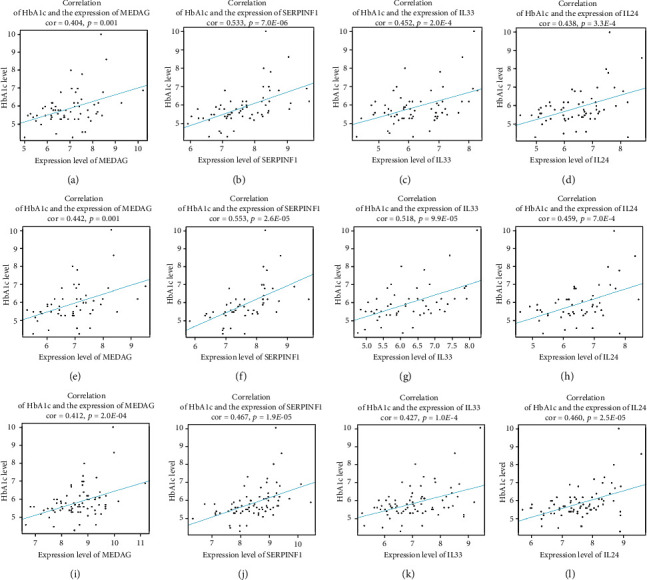
Correlation between real hub genes and HbA1c: (a) correlation between the expression level of MEDAG and HbA1c in dataset GSE41762; (b) correlation between the expression level of SERPINF1 and HbA1c in dataset GSE41762; (c) correlation between the expression level of IL33 and HbA1c in dataset GSE41762; (d) correlation between the expression level of IL24 and HbA1c in dataset GSE41762; (e) correlation between the expression level of MEDAG and HbA1c in dataset GSE38642; (f) correlation between the expression level of SERPINF1 and HbA1c in dataset GSE38642; (g) correlation between the expression level of IL33 and HbA1c in dataset GSE38642; (h) correlation between the expression level of IL24 and HbA1c in dataset GSE38642; (i) correlation between the expression level of MEDAG and HbA1c in dataset GSE50397; (j) correlation between the expression level of SERPINF1 and HbA1c in dataset GSE50397; (k) correlation between the expression level of IL33 and HbA1c in dataset GSE50397; (l) correlation between the expression level of IL24 and HbA1c in dataset GSE50397.

The essential role of a gene in a disease should be easily validated in external datasets if the conclusion is robust, so we reassessed the correlation between gene expression and clinical trait in external datasets obtained from the GEO database (GSE38642 and GSE50397). After the validation, *MEDAG*, *SERPINF1*, *IL33*, and *IL24* still reached the criteria for the real hub gene of T2DM. The correlation between HbA1c and gene expression level is shown in Figure 8.

### 3.8. Validation of a Hub Gene by DEG Analysis

Ninety-two genes were detected as DEGs in the T2DM-related module, including 53 up-regulated genes and 39 down-regulated genes in the T2DM pancreas islet compared with the normal control. The four hub candidate genes were all identified as DEGs. *SERPINF1* was the top 5 upregulated gene, *MEDAG* was the top 7 upregulated gene, *IL33* was the top 17 upregulated gene, and *IL24* was the top 19 upregulated gene. Details of all DEGs are available in Supplement Table 2.

### 3.9. Function Analyses for Real Hub Genes by Single-Gene GSEA

We performed GSEA on *MEDAG*, *SERPINF1*, *IL33*, and *IL24* to explore the role of the genes in the course of T2DM. We performed KEGG enrichment using single-gene GSEA.

**Figure 9:**
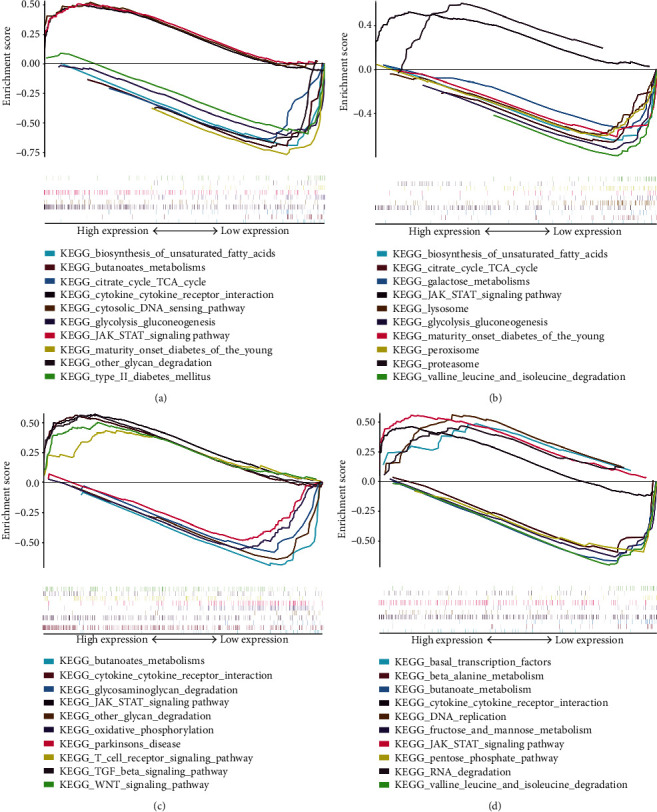
Results of single-gene GSEA: (a) single-gene GSEA of MEDAG; (b) single-gene GSEA of SERPINF1; (c) single-gene GSEA of IL33; (d) single-gene GSEA of IL24.

Results of single-gene GSEA would give some clues about the functions of the four genes. We exhibited the top 10 significant items of single-gene GSEA for *MEDAG*, *SERPINF1*, *IL33*, and *IL24* in Figure 9. The results indicated that the four genes play direct roles in the course of T2DM. For example, *MEDAG* is involved in the signal pathway of “type 2 diabetes mellitus” and “mature onset diabetes of the young.” In “type 2 diabetes mellitus,” the expression levels of *INS* and *GCK* were significantly downregulated. *SERPINF1* may participate in oxidative stress as the change of its expression is involved in the signal pathway of “peroxisome.” *IL24* may function in inflammation. All four genes are significantly related to the metabolism of carbohydrate.

## 4. Discussion

In the present study, we attempted to explore the hub candidate genes in the pathogenesis of T2DM using the combination of bioinformatics analysis and clinical analysis. We conducted WGCNA on the pancreas islet expression profile of T2DM patients and normal control in the dataset of GSE41762 and constructed a weighted coexpression network successfully. Genes were then divided into 16 modules. After identifying a T2DM-related module and a normal-related module, we performed enrichment analyses to explore the function of the genes in the two modules. Then, we constructed a subnetwork consisting of all genes in the T2DM-related module and screened out the top 10 central genes in the module using the MCC method and identified these genes as potential hub genes in T2DM. We then conducted correlation analysis between the expression levels of the top 10 potential hub genes and the level of HbA1c of all samples and confirmed *SERPINF1*, *MEDAG*, *IL33*, and *IL24* as four real hub genes of T2DM and validated the conclusion with two external GEO datasets (GSE38642 and GSE50397). DEG analysis supported that these genes are irregularly expressed in the pancreas islet of T2DM patients. Finally, single-cell GSEA verified and explored the vital roles of the hub genes in T2DM. With all the results above, we conclude that *SERPINF1*, *MEDAG*, *IL33*, and *IL24* are four hub candidate genes in the pathogenesis of T2DM. The functions of *SERPINF1*, *IL33*, and *IL24* in the pancreas islet of T2DM patients have been partly clarified in previous reports. *MEDAG* is a novel hub candidate gene identified by our study.

The pathogenesis of T2DM is still not completely clarified but could be simply come down to two aspects: dysfunction of insulin secretion capacity and peripheral insulin resistance [2]. The dysfunction of insulin secretion is the most significant factor in the mechanism of T2DM and was the focus of our study as we aimed to analyze the expression profile of the pancreas islet. The dysfunction of insulin secretion is caused by the decrease in mass and insulin secretion capacity of islet *β*-cell. Studies have found that T2DM patients have 24% to 65% reduction in the mass of islet *β*-cell [19–21], which may be caused by an increased rate of apoptosis [22, 23] rather than decrease in frequency of cell division or regeneration [24]. The decrease in insulin secretion capacity is the result of multiple factors mainly including dedifferentiation of islet *β*-cell [25], glucose toxicity caused by high level of blood glucose [26], lipotoxicity caused by disorders of lipid metabolism [27], chronic nonspecific inflammation of the pancreas islet [28], and deposition of serum amyloid [29]. Nevertheless, the pathogenesis of T2DM has not been fully explained, and the management of T2DM remains a challenge for physicians. Our study was thus conducted in order to help complete the mechanism study of T2DM by analyzing the mRNA expression level in the pancreas islet. The four hub candidate genes were all positively correlated with the severity of T2DM. However, it is hard to say whether the hub genes are risk factors or compensatory protective factors in T2DM.

Reports have already partly clarified the roles of *SERPINF1*, *IL33*, and *IL24* in T2DM. *SERPINF1* encodes the serpin family of peptidase inhibitors 1, and its most studied member is known as pigment epithelium-derived factor (*PEDF*), which is a multifunctional glycoprotein secreted by adipocytes. Researchers have found that the genetic variant in the gene locus encoding *PEDF* is related to higher risk of T2DM [30] and contributes to the deterioration of T2DM [31]. The protein encoded by *IL33* is a cytokine acting as the ligand of the *IL1RL1/ST2* receptor. It was identified as a protective factor for diabetes mellitus that reduces immune cell infiltration, increases the number of insulin secretion islet *β*-cell, and reduces apoptosis pancreas islet [32, 33]. *IL24* is a member of the *IL10* family, which is significantly overexpressed in the pancreas islet of T2DM. *IL24* is related to endoplasmic reticulum stress, and anti-*IL24* treatment could improve glucose tolerance in T2DM [34].

It is worth mentioning that no reports have clarified the role of *MEDAG* in diabetes mellitus. *MEDAG* encodes a protein named mesenteric estrogen-dependent adipogenesis and is also known as *MEDA-4*. The expression of *MEDAG* in the pancreas islet has been identified by tissue-specific transcriptomics analysis [35]. However, there are relatively few researches concerning the functions of *MEDAG*.

*MEDAG* is located at 13q12.3 and is known to act as an upstream regulator of peroxisome proliferator-activated receptor gamma (*PPARG*), which has been verified as a key regulator in adipose tissue and useful therapeutic target for T2DM. *MEDAG* was newly identified as an adipogenic gene capable of promoting differentiation of preadipocytes into adipocytes. In mature adipocytes, upregulation of *MEDAG* could increase lipid content and promote glucose uptaking. On the contrary, knockdown of *MEDAG* results in decrease in lipogenesis and glucose uptaking [36]. Although the function of *MEDAG* in the pancreas islet has not been clarified, we speculate that it influences the course of T2DM by affecting lipid metabolism and lipotoxicity.

In summary, our research has identified *MEDAG* as a novel hub candidate gene expressed in the pancreas islet in the pathogenesis of T2DM using WGCNA. It was the top 7 upregulated gene according to DEG analysis. The expression level of *MEDAG* is significantly correlated with the level of HbA1c. Three other simultaneously identified hub genes (*SERPINF1*, *IL33*, and *IL24*) have been verified as playing vital roles in T2DM, so that it is reasonable to believe that our result has a high degree of credibility. Further, single-gene GSEA has validated and explored the roles of *MEDAG* in T2DM.

## 5. Conclusions

Our study proposed for the first time that *MEDAG* is a novel hub candidate in the pathogenesis of T2DM through conducting a series of comprehensive bioinformatics analyses on the expression profile of T2DM pancreas islet tissue. *MEDAG* is possible to act as a new focus for molecular mechanism exploration and a novel therapeutic target in the future.

## Data Availability

The expression profile data used to support the findings of this study have been deposited in the GEO database (datasets of GSE41762, GSE38642, and GSE50397).

## Supplementary Materials

Supplementary 1 Supplement Table 1: complete results of GO and KEGG enrichment analyses on the T2DM-related module and normal-related module.

Supplementary 2 Supplement Table 2: details for DEG analysis on the T2DM-related module (including official gene symbols, mean expression levels, log fold change, *p* value, and adjusted *p* value for DEGs).
